# Genomic characterization of the Braque Français type Pyrénées dog and relationship with other breeds

**DOI:** 10.1371/journal.pone.0208548

**Published:** 2018-12-05

**Authors:** Salvatore Mastrangelo, Filippo Biscarini, Marco Tolone, Barbara Auzino, Marco Ragatzu, Andrea Spaterna, Roberta Ciampolini

**Affiliations:** 1 Dipartimento di Scienze Agrarie, Alimentari e Forestali, Università di Palermo, Palermo, Italy; 2 CNR-IBBA, Via Bassini 15, 20133 Milano, Italy; 3 Dipartimento di Scienze Veterinarie, Università di Pisa, V.le delle Piagge 2, 56124 Pisa, Italy; 4 Club Italiano Braque Français Type Pyrénées, Capalbio, GR, Italy; 5 Scuola di Scienze Mediche Veterinarie, University of Camerino, Matelica, MC, Italy; 6 Centro Interuniversitario di Ricerca e di Consulenza sulla Genetica e la Clinica del cane, Matelica, MC, Italy; University of Sydney Faculty of Veterinary Science, AUSTRALIA

## Abstract

The evaluation of genetic variability is a useful research tool for the correct management of selection and conservation strategies in dog breeds. In addition to pedigree genealogies, genomic data allow a deeper knowledge of the variability and genetic structure of populations. To date, many dog breeds, such as small regional breeds, still remain uncharacterized. Braque Français type Pyrénées (BRA) is a dog breed originating from a very old type of gun-dog used for pointing the location of game birds to hunters. Despite the ancient background, the knowledge about levels of genetic diversity, degree of inbreeding and population structure is scarce. This may raise concerns on the possibility that few inbred bloodlines may dominate the breed, and on its future health. The aim of this work was therefore to provide a high-resolution representation of the genome-wide diversity and population structure of BRA dogs, using the 170K genome-wide SNP array. Genome-wide polymorphisms in BRA were compared with those of other worldwide dog breeds. Between-dog relationships estimated from genomic data were very similar to pedigree relationships (Pearson correlation *r*_*g*,*a*_ = 0.92). Results showed that BRA generally presents moderate levels of genetic diversity when compared with the major canine breeds. The estimated effective population size (recent *N*_*e*_ = 51) shows a similar declining pattern over generations as all other dog breeds, pointing at a common demographic history of modern canine breeds, clearly different from the demography of feral wolves. Multidimensional scaling (MDS), Bayesian clustering and Neighbor Joining tree were used to visualize and explore the genetic relationships among breeds, and revealed that BRA was highly differentiated and presented only low levels of admixture with other breeds. Brittany Spaniel, English Setter, Gordon Setter and Weimaraner dogs are the closest breeds to BRA. The exact reason for BRA being so divergent from other dog breeds, based on these results, is not yet clear. Further studies including additional ≪braccoid≫ breeds will be needed to refine the results presented here and to investigate the origin of the BRA breed. Nonetheless, the genome-wide characterization reported here provides a comprehensive insight into the genome diversity and population structure of the Braque Français, type Pyrénées breed.

## Introduction

Modern dog breeds descend from the gray wolf (*Canis lupus*), and through domestication and artificial selection have diversified into a large collection of sub-populations, or breeds, with well-defined morphological and physiological traits [1]. Distinct dog populations have been observed since antiquity and separation into closed breeds during the 19^*th*^ century, together with selection for specific physical attributes, has led to an increase in differentiation among breeds [2]. The breed formation process was associated with severe bottlenecks and a large increase in linkage disequilibrium (LD) [3]. Purebred dogs carry evidence of a high incidence of the “founder effect” (i.e. loss of genetic diversity in subpopulations originating from a small number of ancestors) which leads to longer LD extent than expected [4]. Quantifying the extent of LD is important, as this is likely to have an impact on the success of gene mapping experiments [5, 6]. The genetic variability and the population structure in domestic dog breeds largely depend on the breeders’ decisions and practices. The effective population size (*N*_*e*_) is therefore another important parameter for the assessment of genetic diversity within populations and its development over time.

A comprehensive knowledge of the breeds characteristics is required for the effective management of populations threatened by severe inbreeding and extinction, e.g. genetic rescue by outbreeding with individuals from related subpopulations to augment genetic diversity [7]. This genetic characterization represents the starting point for the development of conservation and breeding programs. With the completion of the sequence of the canine genome [3], the NGS (next-generation sequencing) technology has great potential to increase our understanding of the genomic basis of canid variation, to facilitate the dissection of complex traits through association studies, and to improve the efficiency of selection and conservation programmes [8–10]. LD, *N*_*e*_, inbreeding based on recent methods (i.e. runs of homozygosity) and other population genetics parameters can thus be accurately estimated.

Several studies on the genomic structure and variation within and between dog breeds have been published [8, 10–12]. However, many canine breeds still remain uncharacterized. Braque Français type Pyrénées (BRA) is a dog breed originating from a very old type of gun-dog used for pointing the location of game birds to hunters. The Hunting Treatise “Livre de Chasse” written by the Comte de Foix Gaston Phoebus between 1387—1389 reported the first historical information on a ≪Braccoid≫ type of pointing dogs or ≪chiens d’arret≫ originally from southwestern France in the Pyrénées region and used for hunting wild feathered game, named “Chiens d’oiseaux ou Epagneuls” [13]. The history of BRA is intimately associated with the related breed Braque Français type Gascogne. This latter breed was developed in the Gascogne region of France in the late 1700s from the Old Spanish Pointer and indigenous hounds. However, increasing urbanization led the French people to prefer a smaller dog that could also be kept as a pet in the house. This new need was intercepted by hunters in the Pyrenean region who reportedly crossed Braque Français type Gascogne dogs with smaller pointing and scenting breeds from France, Italy, Spain, Portugal and Germany to create the Pyrénées type. The first breed standards for BRA were written in 1880 and the herdbook was continuously managed by the very same breed club in France until 1920. Braque Français type Pyrénées and type Gascogne were considered two different breeds at that time, and cross-breeding between them was no longer allowed. These breeds have experienced for a long time a relative lack of popularity outside of France. This is rapidly changing, especially for BRA: this breed was introduced in Canada in 1976 and is slowly increasing in numbers in various countries e.g. in Italy. In 2002, the Italian Club Bracco Francese (officially recognized by the ENCI -Italian Kennel Club- in 2006) initiated a selection program and a cynotechnical management for the protection and promotion of this breed in Italy. Additional details on the historical background of BRA and its introduction to Italy can be found in S1 File.

Despite the ancient background, the literature on the genetic characterization of BRA dogs is scarce. A recent study looked into genetic diversity and inbreeding of BRA dogs using molecular markers [14], but investigation of the finer genomic structure of the breed has not yet been reported. It is therefore important to determine baseline genetic data for evolving breeds such as BRA, so to monitor genetic diversity, the effect of artificial selection bottlenecks and to maintain the genetic health of the breed. The aim of this work was to provide a high-resolution representation of the genome-wide diversity and population structure of BRA dogs, using SNP data. Furthermore, genomic polymorphisms in BRA were compared with other worldwide dog breeds. A secondary objective was to determine relationships with a panel of 48 major dog breeds to better understand the origins of BRA. This is the first comprehensive genetic characterization of Braque Français, type Pyrénées dogs from a genome-wide perspective, and of the place of this breed in the broader context of the major canine populations.

## Materials and methods

No experimental studies were conducted on the animals. BRA records were provided by “ENCI” (Ente Nazionale Cinofilia Italiana), the institution that officially manages data for all dog breeds registered in Italy. Blood samples for genotyping were collected by official veterinarians. “Club Italiano Bracco Francese”, whose president is coauthor of the paper, consented for this study to access the blood samples. Genotyping data for all other 49 dog breeds were obtained from the LUPA project (https://eurolupa.org) that made their data publicly available.

### Samples, genotypes and quality control

Blood samples were collected from 48 unrelated individuals of the Braque Français, type Pyrénées dog breed (BRA). These comprised 27 females and 21 males sampled among animals participating in hunting competitions organized by the the Italian Club “Bracco Francese” (C.I.B.F.). Genotyping with the CanineHD Whole-Genome Genotyping BeadChip, containing 173662 SNPs, was performed at the “Dipartimento Scienze Agrarie, Alimentari e Forestali, University of Palermo”, following standard operating procedures recommended by the manufacturer. Genotype data from dog breeds and wolves obtained from the LUPA project ([8]: downloaded from http://dogs.genouest.org/SWEEP.dir/Supplemental.html) were also used (Table 1). Breeds with less than 10 individuals were excluded, leaving 521 dogs from 32 breeds (BRA included) and 13 wolves for the analysis. The 154151 common SNPs across both datasets (BRA and LUPA breeds panel) were filtered as follows: i) markers on unmapped contigs were excluded; ii) only SNPs located on 38 autosomes were retained for further analyses; iii) SNPs with call-rate < 95% and iv) minor allele frequency (MAF) < 1% were removed. Finally, animals with more than 10% of missing genotypes were removed. This resulted in a dataset of 534 animals, 33 breeds (wolves included) and 132281 SNPs that was used to estimate runs of homozygosity (ROH), effective population size (*N*_*e*_) and linkage disequilibrium (LD).

**Table 1:** Summary of canine breeds used in this study: Braque Français and breeds from the LUPA project. Sample size, estimated effective population size (*N*_*e*_) thirteen generations ago, and average inbreeding estimated from ROH (*F*_*ROH*_).

Breed	Abbreviation	N	Ne_13_	F_ROH_
Braque Français	BRA	48	51	0.18
Australian Shepherd	ASH	1		
Beagle	BGL	10	34	0.21
Belgian Tervuren	BET	12	32	0.20
Bernese Mountain Dog	BMD	12	32	0.30
Border Collie	BOC	16	50	0.15
Border Terrier	BOT	25	43	0.33
Boxer	BOX	8		
Brittany Spaniel	BRS	12	49	0.17
Cavalier King Charles Spaniel	CKC	5		
Chihuahua	CHI	2		
Cocker Spaniel	COS	14	44	0.22
Czechoslovakian Wolf Dog	CWD	3		
Dachshund	DAC	12	51	0.15
Dalmatian	DAL	7		
Doberman Pinscher	DOB	25	43	0.37
Elkhound	ELK	12	41	0.17
English Bull Terrier	EBT	8		
English Bulldog	EBD	13	39	0.36
English Cocker Spaniel	ECS	2		
English Setter	EST	12	43	0.20
English Springer Spaniel	ESS	3		
Eurasian	EUR	12	31	0.18
Finnish Spitz	FSP	12	34	0.25
Flatcoated Retriever	FCR	2		
German Shepherd	GSH	12	36	0.31
Golden Retriever	GRE	14	43	0.21
Gordon Setter	GOS	25	65	0.18
Greenland Sledge Dog	GSL	12	53	0.23
Greyhound	GRY	11	33	0.30
Irish Wolfhound	IRW	11	30	0.34
Jack Russell Terrier	JRT	12	52	0.08
Labrador Retriever	LRE	14	50	0.18
Large Munsterlander	LMU	1		
Mops	MOP	2		
Newfoundland	NFD	25	58	0.21
Nova Scotia Duck Tolling Retriever	NSD	23	36	0.24
Rottweiler	RTW	12	36	0.29
Poodles	PDL	17	48	0.18
Samoyed	SAM	2		
Saarloos	SAR	2		
Schipperke	SCI	25	52	0.17
Schnauzer	SCN	3		
Shar Pei	SHP	11	40	0.11
Siberian Husky	HUS	2		
Standard Poodle	STP	12	37	0.17
Terrier	TER	6		
Yorkshire Terrier	TYO	12	51	0.14
Weimaraner	WEI	26	36	0.33
Wolf	WLF	13	49	0.13
*Total*		593		

We further removed SNPs with MAF lower than 5% but using all available breeds (not restricting to breeds with at least 10 individuals), resulting in a dataset of 593 animals, 50 breeds and 123666 SNPs used for multi-dimensional scaling (MDS) of genetic distances, neighbor joining (NJ) clustering and admixture analysis.

For the within-breed analysis of BRA dogs, SNPs with call-rate < 95% and minor allele frequency (MAF) < 5% were removed from the dataset, as well as animals with over 10% missing genotypes (48 dogs and 94065 SNPs remaining).

No experimental studies were conducted on the animals. BRA records were provided by “ENCI” (Ente Nazionale Cinofilia Italiana), the institution that officially manages data for all dog breeds registered in Italy. Genotyping data for all other 49 dog breeds were obtained from the LUPA project (https://eurolupa.org) that made their data publicly available. Blood samples for genotyping were collected by official veterinarians. No animals were sacrificed for the purpose of this study and dogs to be genotyped were volunteered with informed consent from the breeders.

### Genealogical analysis

Genealogical data of the 48 BRA dogs were available. The pedigree file comprised 333 dogs (143 sires, 175 dams), with average depth of 3.6 generations. The numerator relationship matrix (A) of additive genetic relationships between dogs was estimated from pedigree data [15]; the diagonal elements of matrix A are 1 + *F*_*i*_, where *F*_*i*_ are the inbreeding coefficients (probability of identical-by-descent -IBD- alleles at any given locus), with *i* ∈ [1, number of animals] [16]. The R package *pedigree* was used for pedigree calculations [17].

### Estimation of genetic parameters

For the genetic characterization of the BRA dog breed, the following molecular parameters were estimated: i) linkage disequilibrium (LD), ii) effective population size (*N*_*e*_), iii) runs of homozygosity (ROH), and iv) molecular inbreeding. LD between syntenic pairs of SNPs was estimated as *r*^2^ [18]. LD values were grouped into bins based on the base-pair distance between SNPs from the physical map. The average per-bin LD as a function of the base-pair distance was then used to estimate LD decay in BRA dogs and in the other canine breeds.

Recent and historical *N*_*e*_ were estimated based on the relationship between LD (*r*^2^), *N*_*e*_ and recombination rate, as illustrated by Sved [19]. *N*_*e*_ was estimated separately for each breed, and recent vs historical *N*_*e*_ were differentiated based on inter-locus distance.

Runs of homozygosity (ROH) were detected in each animal based on a sliding-window scan of the genome [20]. No explicit pruning was performed based on LD, but a minimum ROH length was set to exclude short ROH deriving from LD. The following criteria were used to define ROH: 50-SNP-long sliding windows with maximum two heterozygous and two missing SNP, and a hit-rate (proportion of overlapping homozygous windows) per individual SNP of 0.05 (default); runs had a minimum length of 50 SNP and 100 kb, a minimum density of 1 SNP every 50 kb, and a maximum gap between consecutive SNP of 100 kb. Based on ROH, molecular inbreeding (*F*_*ROH*_) was estimated per animal by the ratio between the total sum of ROH lengths and the total size of the autosomal genome [21].

### Genetic relationships and population structure

#### Genomic relationships and genetic distances

Within-breed genomic relationships based on SNP genotypes were estimated as described in Yang et al [22]: briefly, additive genetic relationships are estimated between each pair of individual animals, scaling by allele frequencies on a locus-by-locus basis (unlike Van Raden’s method, which scales the matrix of relationships by a constant factor given by twice the sum of allele frequencies [23]). Between-breeds genetic distances were estimated as the complement to one of pairwise IBS (identity-by-state) values (proportion of alleles not IBS between two samples).

#### Population structure

The degree of population substructure was evaluated via the model-based clustering algorithm implemented in ADMIXTURE [24]. The most likely number of populations in the data set (*K*) was estimated through 5-fold cross-validation of the accuracy to assign samples to the correct cluster: the *K* value that minimizes the cross-validation prediction error is then assumed as the most likely. An unweighted neighbor-joining (NJ) tree was constructed using a shared allele index based on the genetic distance matrix estimated from the SNP data.

### Software

Quality control and filtering of the genotypic data, LD estimation, ROH detection, estimation of within- and between-breed genomic relationships were performed using PLINK v. 1.9 [25]. The SNeP tool was used to estimate effective population sizes (*N*_*e*_) [26]. The *R* environment for statistical programming [27] was used for data handling and processing. Specific packages were used to make figures (*ggplot2* [28]) and to construct and visualize the NJ tree (*ape* [29]).

## Results and discussion

The aim of this work was to characterize the genetic variation and population structure of Braque Français, type Pyrénées dogs using genealogical and molecular data: first, to estimate whether the breed still maintains sufficient levels of genetic variability, instrumental in preparing conservation and genetic improvement plans; and second, to place this specific breed in the global context of canine populations, comparing genome-wide polymorphisms in BRA with those of other dog breeds from all over the world.

### Genealogical and genomic relationships

From pedigree data, additive genetic relationships between dogs were estimated. The average inbreeding coefficient ($\bar{F_{i}}$) in BRA was 0.049 (4.9%), with standard deviation 0.051. Similar results were reported by Mortlock et al. [11], which reported a mean inbreeding of 0.047 using pedigree information from 188 Bulmastiff dogs. A previous study on 32 Australian dog breeds reported a mean genealogical inbreeding coefficient ranging from 0 to 0.101 [30], while higher values have been reported in Czechoslovakian Wolfdog [31], in the range 0.19 − 0.23. In our data, 41 out of 48 dogs had *F*_*i*_ > 0, spanning from a minimum of 0.036 (3.6%) to a maximum of 0.25 (25%). The average pedigree inbreeding per generation was rather erratic, going from 0.06 four generations ago to 0.05 in the last available generation in the past, with a maximum at 0.12 (12%) six generations ago, with no clear trend.

Genomic relationships estimated from SNP genotypes were very similar to pedigree relationships, with Pearson correlation of 0.92 (see heatmaps of the two relationship matrices in S1 Fig). Despite this, given the different methodologies behind the calculation of pedigree *F* and molecular *F*_*ROH*_ coefficients [32], estimates of inbreeding levels calculated from genomic data were mildly comparable with those calculated from pedigree data. The correlation between pedigree and molecular inbreeding estimated from runs of homozygosity (*F*_*ROH*_) was 0.48. Wiener et al. [33] reported a Pearson correlation coefficient between pedigree *F* and *F*_*ROH*_ of 0.78. Some studies in livestock species have shown lower correlations between the two inbreeding coefficients; for example, 0.015 in Brazilian Landrace pigs and 0.24 in Brazilian Large White pigs [34]; 0.47 in Danish Jersey cattle and 0.49 in Danish Red Cattle [35]. The moderate correlations reported here could have been influenced by the quality of the pedigree based estimates and by the depth of the available pedigree for BRA. Moreover, pedigree-based estimates assume that there is no inbreeding in the base population and this contributes to the low correlation [34]. More still, the use of genomic data allows Mendelian sampling effects to be estimated more accurately and thus improves the estimates of inbreeding [36]. The use of ROH to estimate inbreeding has been applied for some years to several livestock species e.g. [37, 38], more recently to dogs [11, 14, 33]. A strong correlation between pedigree *F* and *F*_*ROH*_ has been reported by several authors –here examples in cattle [21, 39]–, suggesting that the extent of genome in ROH may effectively be used to infer aspects of recent population history even from relatively few samples.

### Runs of homozygosity, effective population size and linkage disequilibrium

Reduced genetic diversity can indicate the occurrence of phenomena like selective breeding, founder effects or population bottlenecks: genetic diversity is therefore directly relevant to maintain a healthy breed and reduce morbidity from inherited disorders in dogs [40]. We analyzed ROH, *N*_*e*_ and LD for BRA and all dog breeds with more than 10 genotyped individuals from the LUPA database, to assess their inbreeding levels and to compare genetic parameters with BRA.

**Fig 1:**
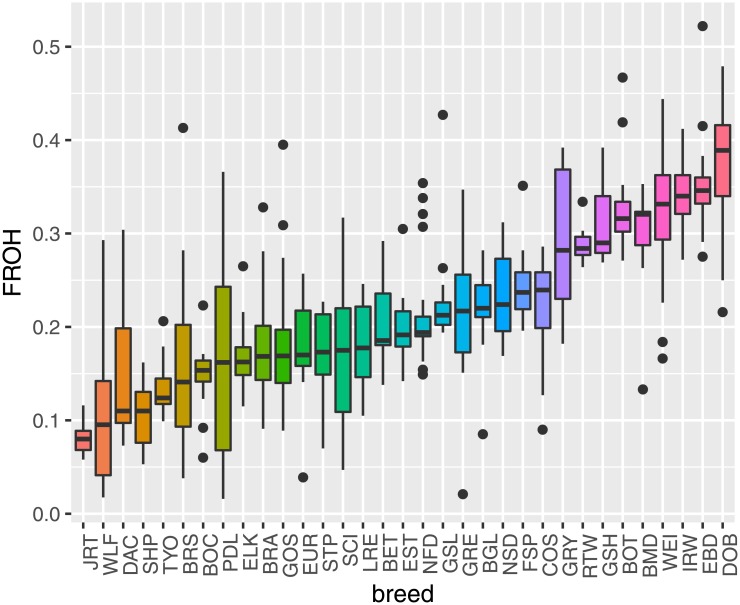
Distribution of *F*_*ROH*_. Boxplots of individual *F*_*ROH*_ values for all dog breeds with sample size ≥ 10, ordered by increasing median *F*_*ROH*_.

The ROH coverage of the genome differs considerably among breeds (Fig 1). The highest mean values of *F*_*ROH*_ across all breeds were observed in Doberman Pinscher (DOB) (0.371) and English Bulldog (EBD) (0.357). Other breeds generally showed moderate *F*_*ROH*_ values, like BRA (0.176 ± 0.049), in a range from 0.091 to 0.328. Recently, similar average values (0.17 ± 0.02) were reported for the Czechoslovakian Wolfdog [31]. The breeds that showed the lowest levels of inbreeding included Jack Russell Terrier (JRT) (0.080) and Shar Pei (SHP) (0.106). Mortlok et al. [11] reported *F*_*ROH*_ ranges from 0.061 in Jack Russell Terriers to 0.151 in Bulldogs, while Dreger et al. [10] reported maximum average *F*_*ROH*_ = 0.39 in Pharaoh Hounds and minimum *F*_*ROH*_ = 0.03 in Mastino Abruzzese. The BRA breed shows therefore inbreeding levels well within the range detected in other dog breeds.

As detailed previously [41], the effective population size (*N*_*e*_) of a real population can be defined as the size of a hypothetical ideal population that would result in the same amount of genetic drift as in the (actual) current population. *N*_*e*_ is an important population parameter that helps explain how populations have evolved, and it can be used to improve the understanding and modeling of the genetic architecture underlying complex traits [42]. The genomic approach to estimating *N*_*e*_ is promising, especially in situations where the depth of the available pedigree data is low and pedigree-based estimates are thus likely to be inaccurate.

**Fig 2:**
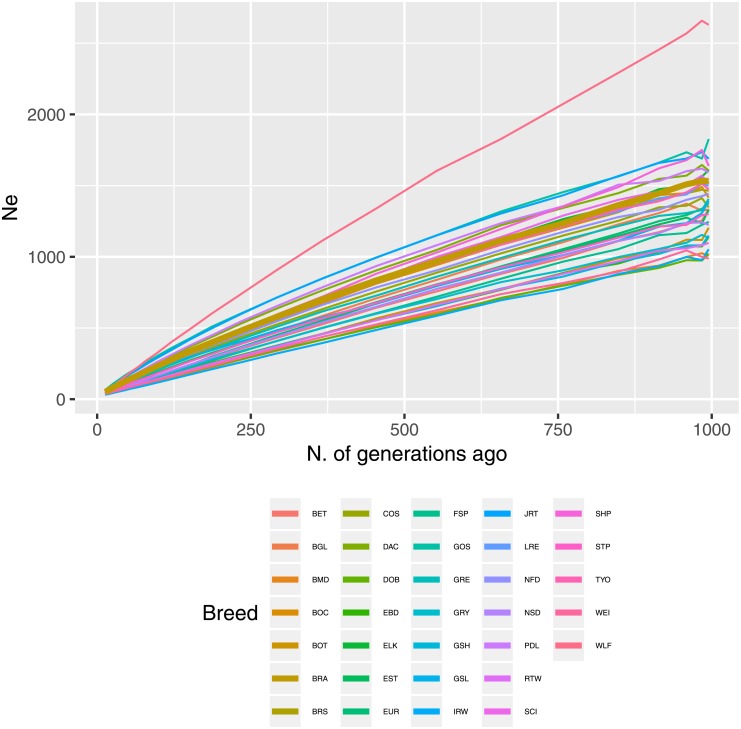
Average estimated *N*_*e*_ over time. Per-breed estimated *N*_*e*_ values along number of generations in the past (from left to right). The thicker line represents the BRA breed.

Estimated *N*_*e*_ at each of *t* ∈ *T* generations in the past (*T* = [13, 995]) are shown in Fig 2. As expected, a progressive decrease in *N*_*e*_ over generations was observed. The decreasing trend is likely due to the progressive selection and the within-breed use of top performing dogs. *N*_*e*_ estimates showed that 13 generations ago the effective population size was < 70 for all breeds. Different estimates have been reported by Dreger et al. [10] with recent *N*_*e*_ values ranging from 71 (Neapolitan Mastiff) to 303 (Saluki). Ancestors of the contemporary Wolf (WLF) exhibited considerably larger *N*_*e*_ values. In the present study, recent (13 generations ago) and past (995 generations ago) *N*_*e*_ in BRA were estimated to be 51 and ∼ 1530, respectively. The breeds that showed the lowest recent *N*_*e*_ value were Irish Wolfhound (IRW) (*N*_*e*_ = 30) and Eurasian (EUR) (*N*_*e*_ = 31).

As the *N*_*e*_ decreases, there is an increased risk of mating genetically related individuals. A population with *N*_*e*_ under 50 can be considered at risk from the negative effects of inbreeding depression [43]. This may be especially true for the survival of a wild freely breeding population, but may not be the case where the survival of the species is bolstered by human intervention. For instance, the California Condor declined to 23 individuals worldwide in zoos and captivity by 1982 and subsequently saved from extinction by captive breeding (but at a tremendous financial cost and very strict management of habitat [44]). Several other species that had gotten to even lower numbers have been saved in captivity [45]. Therefore, the estimated effective population size for BRA, though close to the theoretical critical threshold of *N*_*e*_ = 50, may imply that the viability of the breed is not compromised if the population is well managed. Additionally, while qualitative *N*_*e*_ estimates (shape and pattern of *N*_*e*_ over generations) tend to be rather robust, this is not true for quantitative *N*_*e*_ estimates. *N*_*e*_ point estimates are highly sensitive to parameters like sample size, LD extent and metric, and binning system (inter-locus distances), and their values may change dramatically between studies, especially for more than 4 ⋅ *N*_*e*_ generations in the past [26].

**Fig 3:**
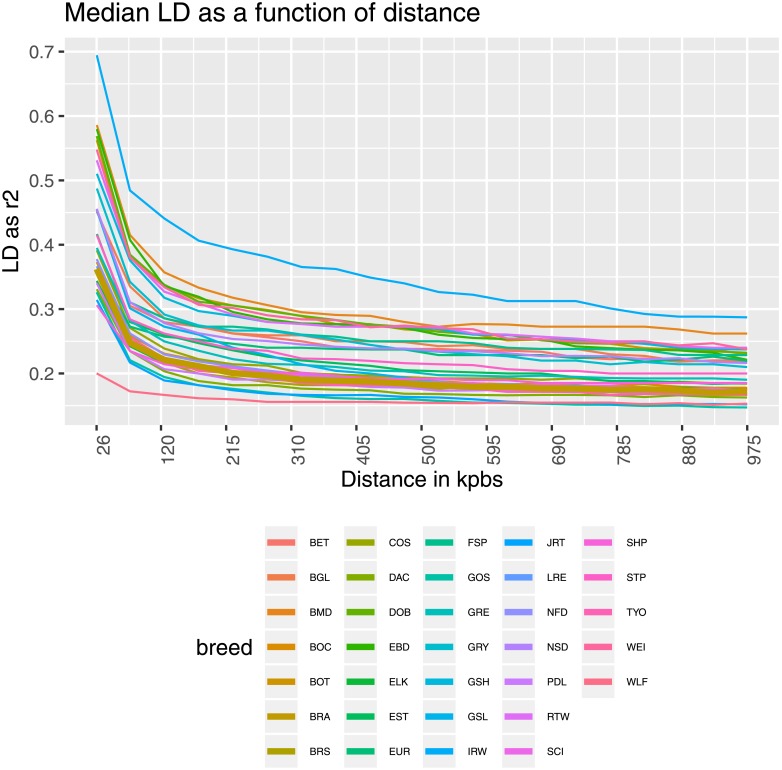
LD decay as a function of bps distance. Per-breed LD decay (measured as *r*^2^) as a function of inter-marker distance in bps. The thicker line represents the BRA breed.

To predict the extent of LD the *r*^2^ statistic was chosen over the *D*′ estimator. *D*′ estimates tend to be inflated with small sample sizes or at low haplotype frequencies [46]. *r*^2^ is generally thought to be the best suited measure of LD for biallelic markers and to avoid the influence of small sample size [47]. The LD decay—as *r*^2^—over all 38 autosomes together in each breed is displayed in Fig 3. Genome-wide average LD decreased with increasing genomic distance for all breeds. Large differences between the breeds were observed. The most persistent LD over distance was observed in the Irish Wolfhound (IRW). Wolf (WLF) populations had the fastest LD decay, as expected for an ancestral outbred group [9] with high *N*_*e*_ values. However, for distant markers (650–1000 kb) the LD extent stretched longer in wolves (WLF) than in Gordon Setter (GOS). As a general pattern, breed differences decreased as between-marker distance increased. BRA dogs showed intermediate LD decay, with the average *r*^2^ falling below 0.20 after 220 kb. Stern et al. [6] identified different inter-marker distances at which the average *r*^2^ dropped below 0.25 in three dog breeds: from 100-200 kbps in Newfoundland dogs, to 200-500 kbps in Golden Retrievers, to 0.5–1 Mb in Rottweilers. Average *r*^2^ < 0.1 for markers over 820 kbps apart was reported in UK Labrador Retrievers [33]. The variation observed in our study confirms that even breeds that are of similar size, and have similar genetic architecture, are not effectively the same in terms of their extent of LD.

### Genetic relationship and population structure among all dog breeds

Ascertainment of population structure and genetic relationships between animals have proven useful in conservation and management practices. Multidimensional scaling (MDS), Bayesian-model based clustering, and Neighbor Joining were used to visualize and explore the genetic relationships among breeds.

**Fig 4:**
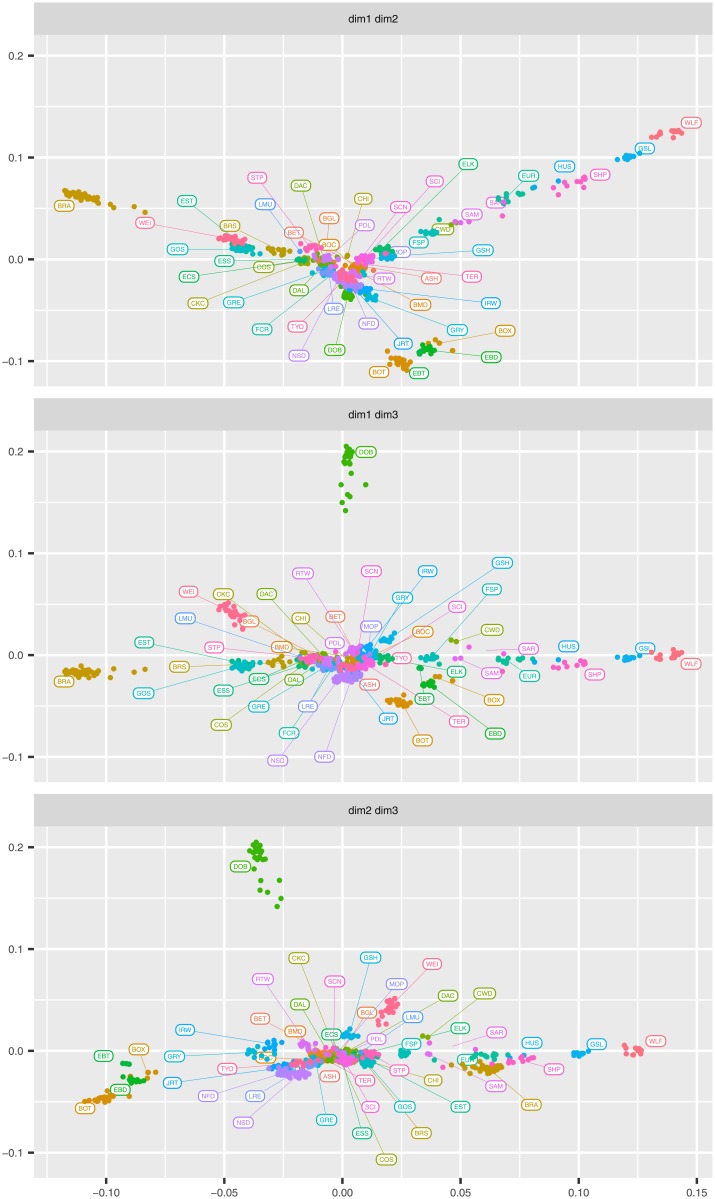
Multidimensional scaling (MDS) plot of marker-based genetic distances among dog breeds. Visualization of genetic distances based on SNP data among BRA and 49 other breeds from the Lupa project. The first three MDS dimensions are shown in pairwise plots. Breed labels are as in Table 1.

**Fig 5:**
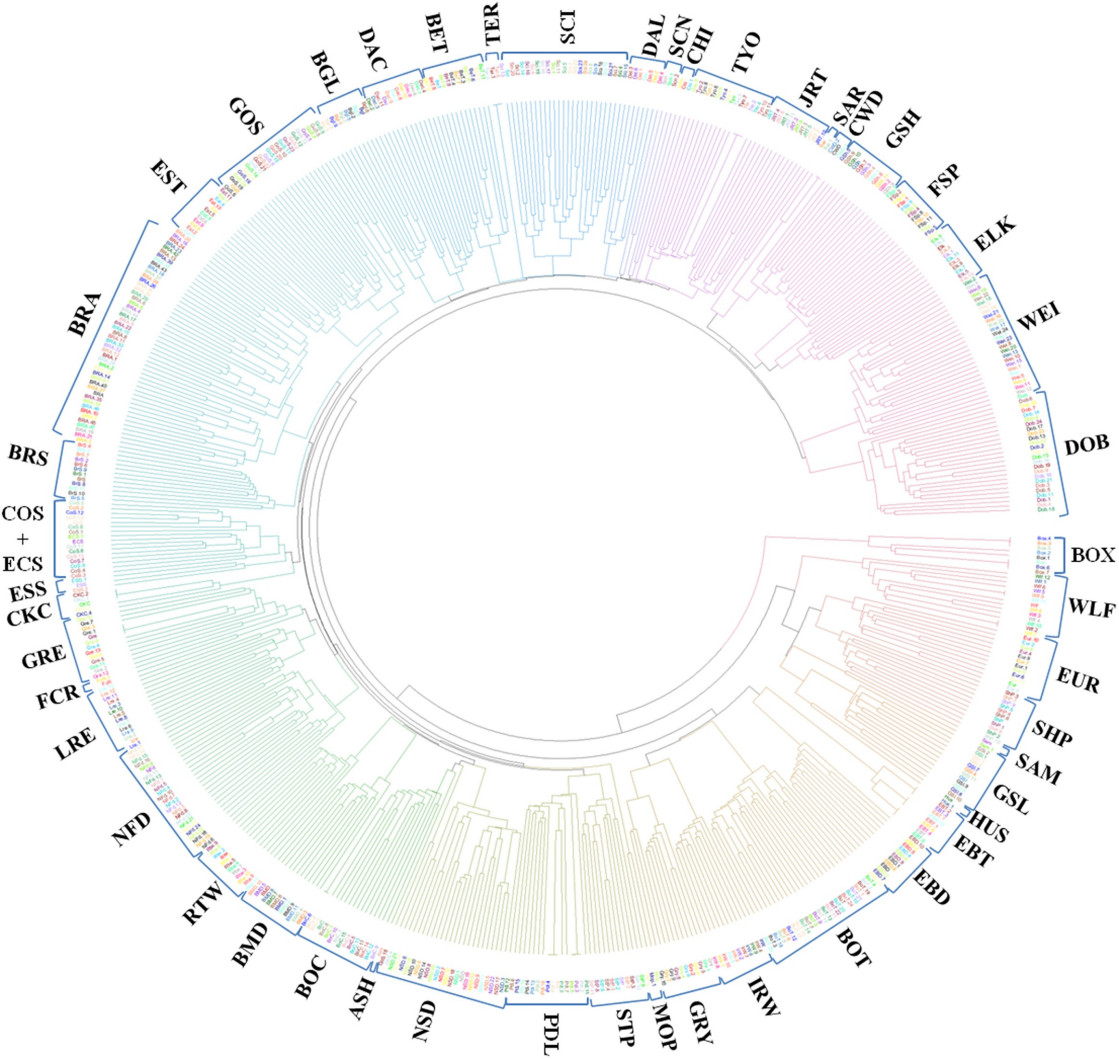
Neighbor-joining (NJ) tree. Neighbor-Joining tree visualization of genetic distances based on SNP data among BRA and 49 other breeds from the Lupa project.

Overall, BRA appears genetically distinct from the other dog breeds involved in this study. BRA is thought to be closely related to a number of European pointing breeds. The only ≪Braccoid≫ continental breed belonging to the “Federation Cynologique Internationale” (FCI http://www.fci.be/en/) Group 7 “Pointing Dogs group and Setters” present in the LUPA Database is the Weimaraner (WEI). BRA is thought to be closely related to WEI with which shares structural characteristics and the hunting attitude for wild feathered game. This is supported by the first two dimensions of the MDS plot (Fig 4, top). However, the NJ tree showed close relationships between BRA, English Setter (EST), Brittany Spaniel (BRS) and Gordon Setter (GOS), and a weaker relationship between BRA and WEI (Fig 5). At first puzzling, these results appear clearer when looking at additional dimensions from the MDS plot (Fig 4, middle, bottom): in the multi-dimensional hyperspace (3-D here), BRA and WEI clustered farther away from each other, while EST, GOS and BRS were still relatively closer to BRA. Despite having a completely different anatomical structure, these latter breeds share similar hunting attitude and modalities toward feathered game with BRA. We can hypothesize that selection for similar hunting behaviour, even in breeds of different geographical origin and with dissimilar anatomical features, is presumed to have contributed to the shaping of a similar genetic background. These results are consistent with previous studies that showed that dog breeds with similar hunting skills, like setters and spaniels, tend to group together genetically [8, 11].

As for the other breeds from the LUPA dataset, the MDS plot analyses (first two axes) revealed three main clusters. In particular, the majority of “modern” European dog breeds were clustered together in the centre of plot and they could not be easily distinguished from each other, suggesting a relatively high gene flow between them. East Asian (Shar-Pei (SHP)), Arctic (Greenland Sledge Dog (GSL), Siberian Husky (HUS) and Samoyed (SAM)) breeds, together with the Czechoslovakian Wolfdog (CWD), Eurasian (EUR), Saarloos (SAR) and Finnish Spitz (FSP) breeds, formed a distinct cluster from most European breeds. Some of these divergent breeds (SHP and HUS) have been pinpointed and collectively termed “ancient breeds” [1]. Fig 4 also showed that these breeds appear to be closer to wolves, which clustered independently, in agreement with previous findings [8–10]. As a matter of fact, the observed lack of admixture with wolves is consistent with the strict breeding regimes of canine breeds, since modern breeds are the products of highly controlled breeding practices [1]. Furthermore, the second MDS axis seems to separate Border Terrier (BOT), Boxer (BOX), English Bull Terrier (EBT) and English Bulldog (EBD) from other European breeds. As already pointed out in previous studies based on medium density chip [1], a lack of a clear geographical gradient in molecular variation was observed.

**Fig 6:**
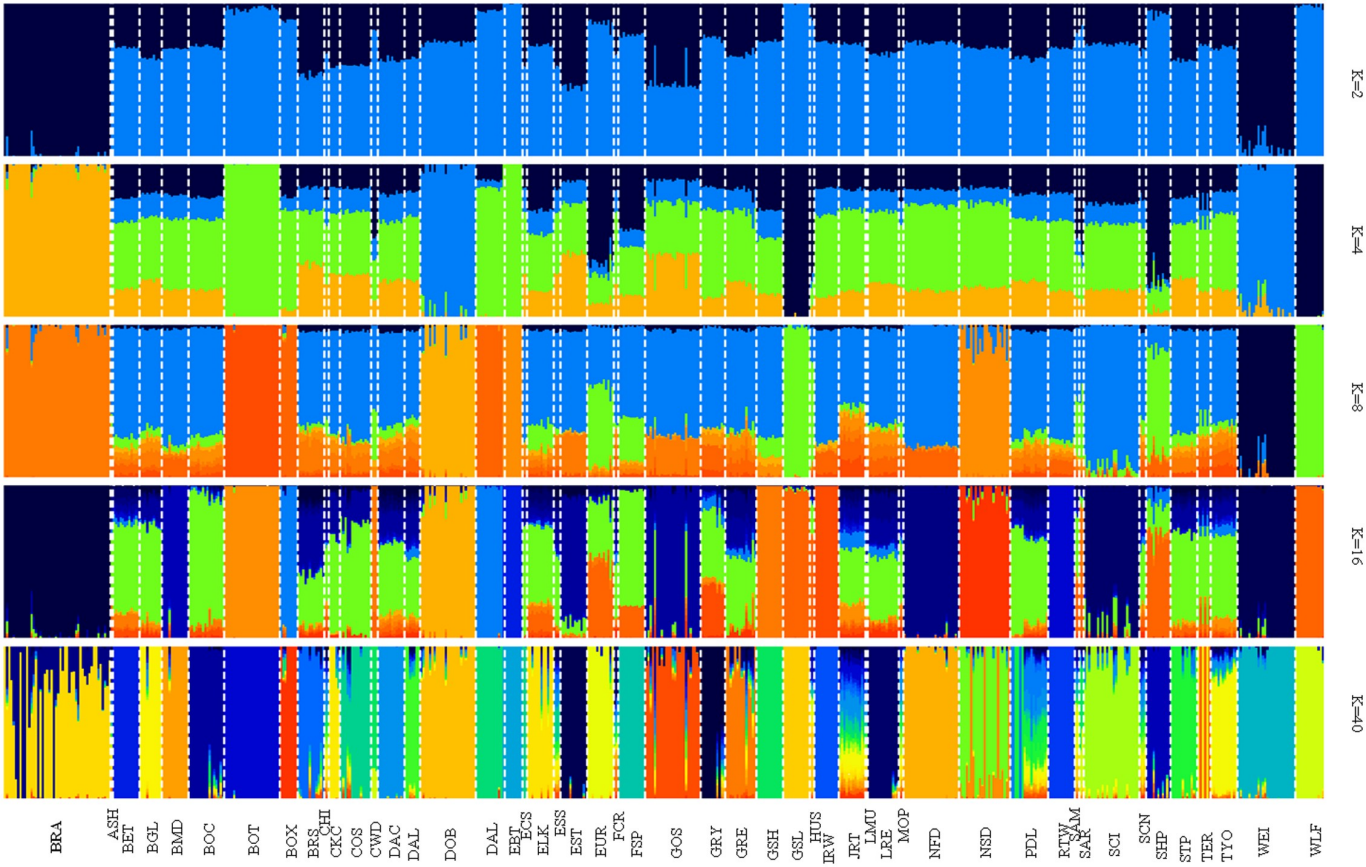
Population structure of BRA and LUPA dog breeds. Bayesian clusterings from ADMIXTURE with varying values for the parameter K (*K* ∈ 2, 4, 8, 16, 40).

All dogs were assigned with the highest probability to the breed from which they were sampled: as reported by Vaysse et al. [8] this shows that modern breeds are essentially closed gene pools that originated via population bottlenecks. The genetic ancestry of dog breeds was further examined by varying the number of ancestries (*K*) in a Bayesian clustering algorithm (Fig 6). The first clustering (*K* = 2) reflected the separation of Wolves (WLF) from domestic dog breeds. At *K* = 4, we observed a strong genetic differentiation between BRA and the remaining dog breeds and this was also found at higher K-values. At the same K-value, Border Terrier (BOT), English Bull Terrier (EBT) and Greenland Sledge Dog (GSL) showed distinctiveness from the other breeds.

The most probable number of populations present in the total sample, as suggested by the ADMIXTURE cross-validation procedure was *K* = 40. At this K value, generally a distinct cluster is observed for each dog breed, although with some differences. As a matter of fact, some breeds (Jack Russell Terrier (JRT), Poodles (PDL)) together with BRA showed less distinct clusters. This result is in line with the historical information available on BRA, suggesting that this breed may be a mixture of European (Spanish) Pointer dogs.

When K was increased, breeds were progressively assigned to separate clusters. In general, the ADMIXTURE analysis followed the affinities among breeds found in the MDS plot and NJ tree. BRA was highly differentiated and presented only low levels of admixture with other breeds. This could be related to a combination of low population size and isolation from other breeds. Very little is known with certainty about the origin of BRA, as this breed was developed before written records of dog breeding began to be kept. What is known is that the breed was developed in France before the late 1700s, and that it was primarily used for tracking, pointing at and retrieving game. Selection programmes began in the last decades of the 1800s, as was the case for most modern dog breeds, from a population with ancestral morphology (a rather heavy Braque type) with the aim of developing a lighter type. Over time selection led to two different types characterized by different morphological aspects and consequently, different functions.

The exact reason for BRA being so divergent is not yet clear. Based on the results here reported, none of the major canine breeds appears to have played a major role in the ancestry of BRA, supporting its origin from old Spanish Pointer dogs which goes far back in time. Further studies including other ≪Braccoid≫ dog breeds, in particular the Braque Français type Gascogne, would be relevant to unravel the origin of BRA. Nonetheless, the genome-wide characterization reported here provides a comprehensive insight into the genomic diversity of Braque Français, type Pyrénées dogs.

## Conclusion

This is the first study to estimate the population structure of Braque Français type Pyrénées dogs from a genome-wide perspective. From a within-breed perspective, BRA shows lower than average molecular inbreeding and extent of LD, and higher than average effective population size, when compared to the other dog breeds considered in the study. From a between-breeds perspective, BRA displayed clear genetic differentiation, thereby confirming the available historical information which suggests that BRA is a mixture of older European Pointer dogs.

Monitoring inbreeding and genetic variability is important to reduce the incidence of heritable diseases and maintain overall fitness of the breed. Therefore, considering that genetic diversity is an intrinsic factor that influences the adaptive capacity and resilience of populations, the outlined genomic approaches represent useful tools for the implementation of carefully planned selection and mating strategies within breeds. These results further suggested that the combined use of genomic and pedigree data may be a valid option for the selection of lineages that minimize the inbreeding rate per generation. Breeders should be aware of the risks related to excessive inbreeding and of the opportunities offered by genomics, so to design breeding systems that foster and maintain genetic variation in the BRA breed.

## Supporting information

S1 File History of BRA and introduction to Italy. A few elements on the history of the Braque Français type Pyrénées canine breed, and the directional selection practiced in Italy.
(PDF)

S1 Fig Kinship matrices for Braque Français, type Pyrénées dogs. Heatmap of kinship matrices in BRA estimated from pedigree data or from molecular marker data.
(PDF)
