# Supplementary material for: Genomic characterization of the Braque Français type Pyrénées dog and relationship with other breeds
Source: PLoS One. 2018 Dec 5;13(12):e0208548. doi: 10.1371/journal.pone.0208548 (PMC6281230; doi:10.1371/journal.pone.0208548)
Supplement: S1 File — A few elements on the history of the Braque Français type Pyrénées canine breed, and the directional selection practiced in Italy. (PDF) [file pone.0208548.s001.pdf]

Genomic characterization of the Braque Français type Pyrénées dog and relationship with other breeds

Salvatore Mastrangelo[1], Filippo Biscarini[2]* et al.,

1 Dipartimento di Scienze Agrarie e Forestali, Università di Palermo, Palermo, Italy

2 CNR-IBBA, Via Bassini 15, 20133 Milano, Italy

*E-mail: filippo.biscarini@ibba.cnr.it

The history of the Braque Français type Pyrénées, and the directional selection practiced in Italy

The Hunting Treatise entitled "**Livre de Chasse**" 1387 – 1389 (http://classes.bnf.fr/phebus/livre/index.htm) written by Count of Foix Gaston Phoebus, reports the first historical and iconographic information concerning a «Braccoid» type of pointing dog or «chiens d'arret» originating in the south-west of France, in a region corresponding to the present Pyrénées region, where the the Shire of FOIX was located (Figure 1).

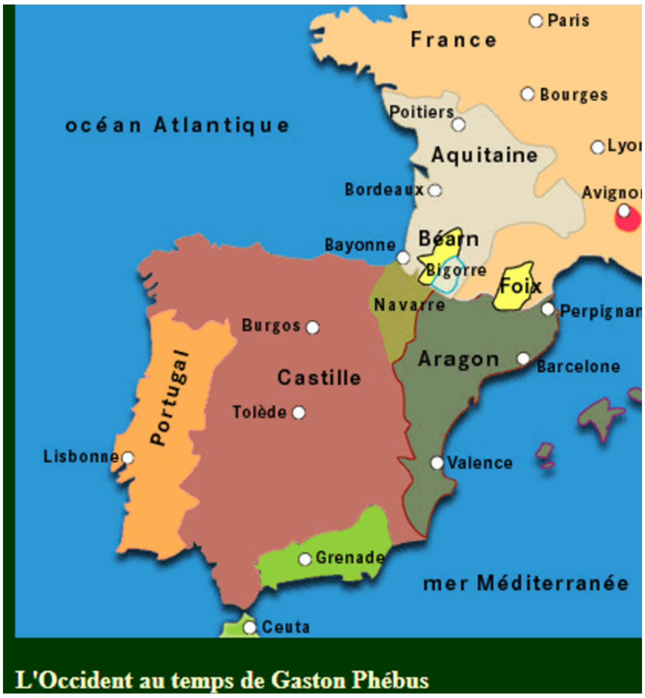

Figure 1: Shire of FOIX

This breed, whose graphic representation in the form of artistic paintings is taken from Folio 40v (Livre de Chasse Folio 40v: http://expositions.bnf.fr/phebus/grands/c16_616.htm; see Figure 2), was used for feathered game hunting, and Gaston Phoebus named this breed "Chiens d'oiseaux ou Epagneuls".

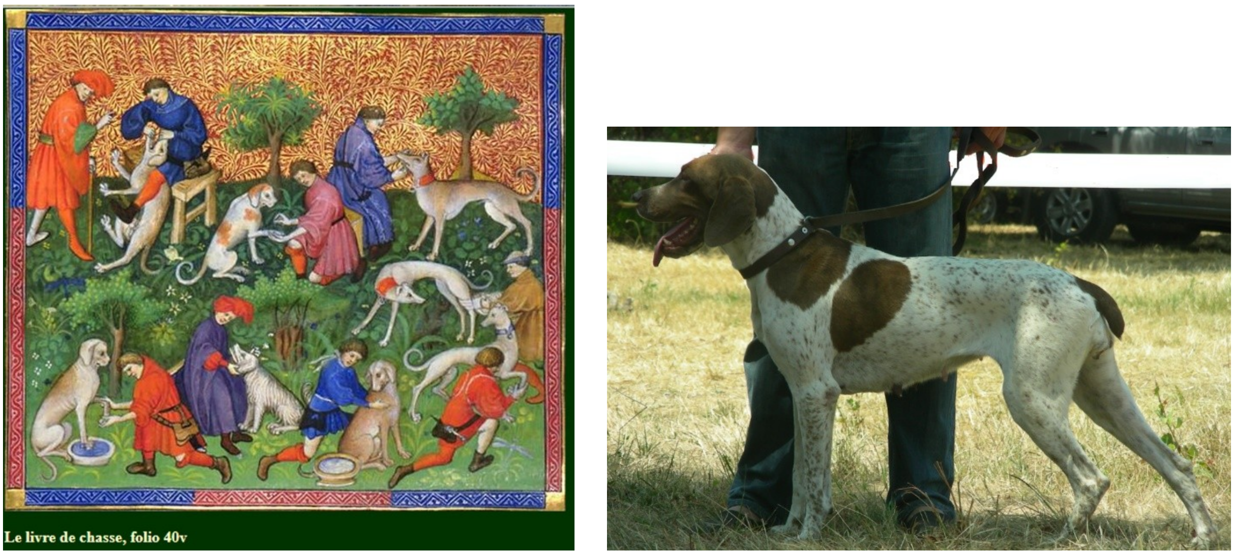

Figure 2: paintings representing: left, ancient specimens of Chiens d'oiseaux ou Epagneuls; right, the modern Braque Français type Pyrénées dog.

In France, artificial selection was carried out with precise criteria from the last decades of the 1800s, as was the case for all modern dog breeds. The work of selection started from a population with an ancestral morphology (a rather heavy Braque) orientating towards a lighter morphology. Over the centuries the selection has determined the creation of two different typologies characterized by different morphological aspects and consequently, different functional aspects:

1. the Braque Français Type Pyrénée, originally from the central area of the Pyrénées, of smaller size with a light skeletal structure and a neurotic temperament
2. the Braque Français Type Gascogne, originating in the south-west of France, of a more imposing size with a heavier skeleton and less neurotic temperament.

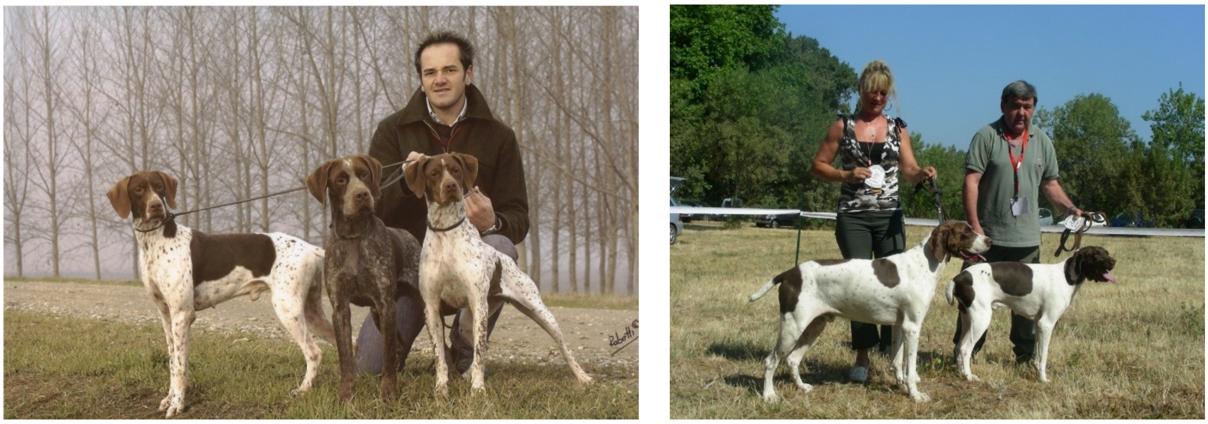

Figure 3: Braque Français Type Pyrénée, left; Braque Français Type Gascogne, right.

Beginning in 2002, the presence of the Braque Français Type Pyrénée in Italy was initially small, with very limited imports of sires and dams and few litters produced by Italian breeders. In 2006, the Italian Club Bracco Francese (CIBF) was Founded and accredited by ENCI “Ente Nazionale per la Cinofilia Italiana” (Italian Kennel Club). CIBF initiated a careful selection programme with the aims of protection and promotion of the breed. The selective objective aims at obtaining Braque Français Type Pyrénée dogs well distinguished from the Type Gascogne, and at the same time far from the peculiarities that characterize the **English Pointer** and **German Pointer**, preserving the somatic aspects concerning the points of the region of the head and of the whole trunk. The collaboration with the French Club of Braque Français Type Pyrénée has allowed to identify in France the best breeders and their products and to acquire at the same time a precise zootechnical knowledge that allowed to start the importation in Italy of sires and dams belonging to bloodlines defined as "pure" that did not have ancestors of the Gascogne type. This guarantees the obtainment of subjects of Braque Français Type Pyrénée that are most interesting to the Italian breeders. This is how selection for this breed began in Italy, which benefited from a close collaboration between the members of the CIBF, who wisely considered the indications provided by the CIBF Technical Committee, both on the use of sires and dams born in Italy and on the use of imported French sires and dams. CIBF was also officially assigned the task of distributing puppies in Italy, trying to guarantee a widespread and diversified presence throughout the peninsula, without concentration of subjects of the same litter in the same territory to facilitate the choice of male and female breeders for future mating. A thorough check of the results obtained with the implementation of the described selective schemes has always been implemented thanks to the evaluation tools adopted by CIBF and consisting of functional checks, (work tests) and morphological checks (gathering and exhibitions), organized directly by CIBF, with the objective of the morpho-functional evaluation of parents and young generations.

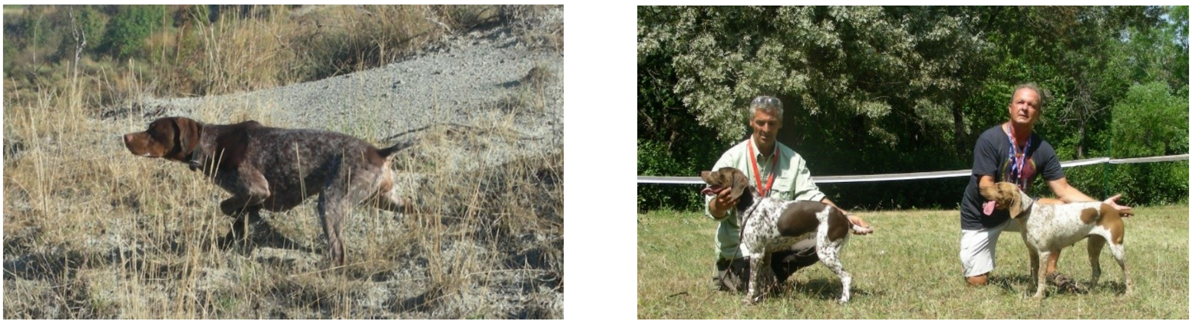

Figure 4: work tests left; morphological checks, right.

This management of the breed by the CIBF has allowed to obtain a sufficiently broad genetic base and has been constantly monitored from a selective and cynotechnical point of view, which over time has allowed both to preserve the purity of the phenotypic characteristics and to maintain the aptitude characteristics of Braque Français Type Pyrénée dogs. Today, after fifteen years of careful and rigorous selection work, we can speak of a true "subpopulation" of the Braque Français Type Pyrénée in Italy.
