# Supplementary material for: Genomic characterization of the Braque Français type Pyrénées dog and relationship with other breeds
Source: PLoS One. 2018 Dec 5;13(12):e0208548. doi: 10.1371/journal.pone.0208548 (PMC6281230; doi:10.1371/journal.pone.0208548)
Supplement: S1 Fig — Heatmap of kinship matrices in BRA estimated from pedigree data or from molecular marker data. (PDF) [file pone.0208548.s002.pdf]

Genomic characterization of the Braque Français type Pyrénées dog and relationship with other breeds

Salvatore Mastrangelo[1], Filippo Biscarini[2*] et al.,

1 Dipartimento di Scienze Agrarie e Forestali, Università di Palermo, Palermo, Italy
2 CNR-IBBA, Via Bassini 15, 20133 Milano, Italy
*E-mail: filippo.biscarini@ibba.cnr.it

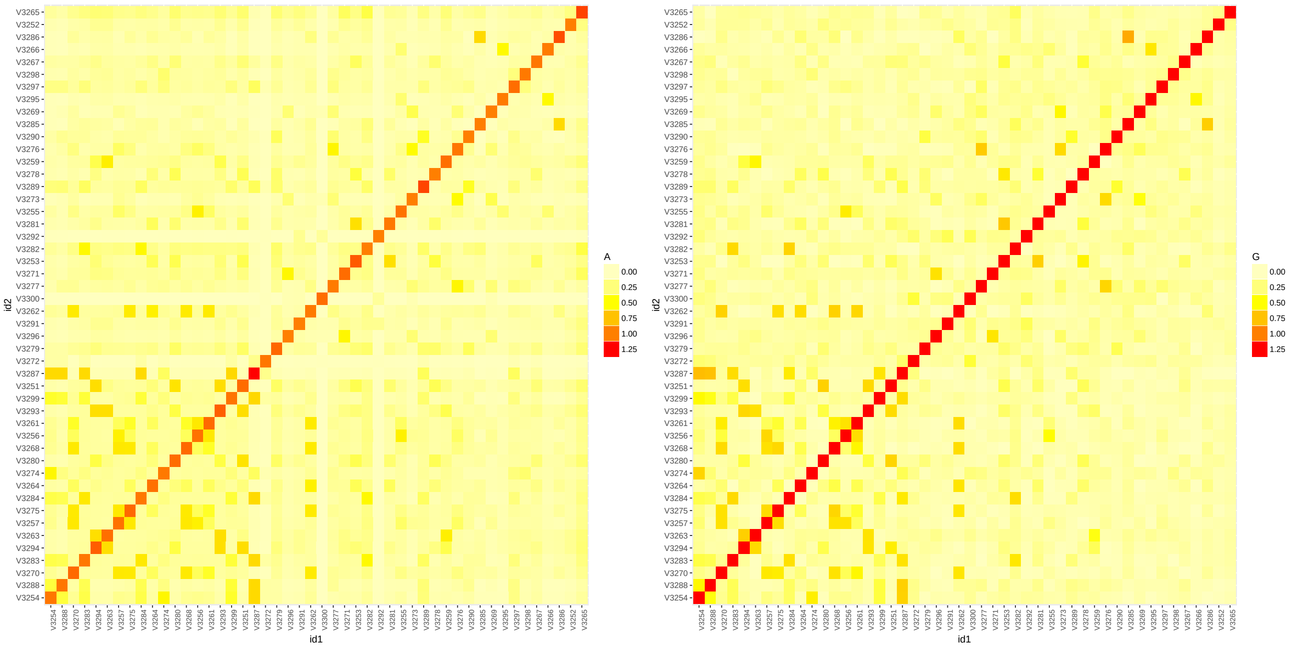

S1 Fig: **Genealogical and molecular kinship matrices.** Heatmaps of kinship matrix based on pedigree data (left) and SNP marker data (right) for 48 the Braque Français, type Pyrénées dogs sampled for this study
